# OmniChange: The Sequence Independent Method for Simultaneous Site-Saturation of Five Codons

**DOI:** 10.1371/journal.pone.0026222

**Published:** 2011-10-19

**Authors:** Alexander Dennig, Amol V. Shivange, Jan Marienhagen, Ulrich Schwaneberg

**Affiliations:** Lehrstuhl für Biotechnologie, RWTH Aachen University, Aachen, Germany; University of Massachusetts Medical School, United States of America

## Abstract

Focused mutant library generation methods have been developed to improve mainly “localizable” enzyme properties such as activity and selectivity. Current multi-site saturation methods are restricted by the gene sequence, require subsequent PCR steps and/or additional enzymatic modifications. Here we report, a multiple site saturation mutagenesis method, OmniChange, which simultaneously and efficiently saturates five independent codons. As proof of principle, five chemically cleaved DNA fragments, each carrying one NNK-degenerated codon, were generated and assembled to full gene length in a one-pot-reaction without additional PCR-amplification or use of restriction enzymes or ligases. Sequencing revealed the presence of up to 27 different codons at individual positions, corresponding to 84.4% of the theoretical diversity offered by NNK-degeneration. OmniChange is absolutely sequence independent, does not require a minimal distance between mutated codons and can be accomplished within a day.

## Introduction

Directed evolution and rational design are two complementing and often synergistic approaches for tailoring biocatalyst properties to application demands [1], [2]. Directed evolution improves enzyme properties through iterative rounds of random gene diversification, screening and selection and is especially useful for improving properties when reliable structural models are not available or rationally not well understood [3]. Activity and selectivity are two enzyme traits that have successfully been improved through rational and semi-rational protein engineering by employing focused mutagenesis to generate mutant libraries [4], [5]. The semi-rational CASTing approach employs a Mutagenic Plasmid Amplification method [6] which was successfully used for improving enantioselectivity of an epoxide hydrolase through iterative rounds of multi site-saturation mutagenesis and subsequent screening by targeting residues located in the substrate binding pocket [7].

Efficient single site-saturation mutagenesis methods are Codon Cassette Mutagenesis, MOD-PCR (Mutagenic Oligonucleotide-Directed PCR Amplification), OEP (Overlap Extension PCR) and Mutagenic Plasmid Amplification [8], [9], [10], [11], [12]. The principle of Mutagenic Plasmid Amplification is probably today the most commonly used method for site-directed and site-saturation mutagenesis and was commercialized by Stratagene as a kit (QuikChange Site-Directed Mutagenesis Kit, La Jolla, CA, USA). In Iterative Saturation Mutagenesis (ISM) the QuikChange method was successfully extended to subsequent cycles of saturation mutagenesis and screening for improved thermal resistance of a lipase (Lip A) from Bacillus subtilis [13].

Multi codon mutagenesis methods differ in the number of simultaneously mutated positions, the number of PCR steps and application of DNA modifying enzymes (Table 1). POEP (Polyacrylamide Gel Electrophoresis-mediated Overlap Extension Polymerase Chain Reaction; [12]) relies on the principle of overlap extension PCR with two main steps (fragment generation PCRs & assembling PCR) and is limited by decreased assembling PCR efficiency with increasing number of fragments [14]. Seyfang and Jin [15] developed a multi site-directed mutagenesis method requiring several phosphorylated mutagenic primers that simultaneously anneal to the template DNA, get linear amplified with T4 DNA polymerase, followed by ligation in vitro with T4 DNA ligase and a final amplification via PCR. The main challenge of the method from Seyfang and Jin lies in the simultaneous non-preferentially annealing of all mutagenic primers requiring similar thermodynamic properties (GC-content, annealing temperature). Applying this multiplex-PCR approach becomes therefore more and more difficult with increasing number of targeted positions [16], [17]. The commercially available QuikChange Multi Site-Directed Mutagenesis Kit (Stratagene, La Jolla, CA, USA), follows a similar strategy by using a polymerase/ligase blend for ligation of up to five mutated (site-directed mutagenesis) and phosphorylated ssDNA fragments in a multiplex DNA-amplification step [18]. Conceptually interesting is also the ISOR-method (Incorporating Synthetic Oligonucleotides via Gene Reassembly) in which 45 codons could be targeted simultaneously by mutagenic primers for achieving an average of 5.6 saturated codons per gene [19].

**Table 1:** Overview of methods and strategies for focused mutagenesis on multiple positions.

Performance	POEP [12]	OD SPM [15]	ISOR [19]	Iterative CASTing [7]	QuikChange Multi Site-Directed Kit [18]	OmniChange (This study)
*SDM* * */SSM* **	+/−	+/−	−/+	−/+	+/+	−/+
*Simultaneously mutated sites*	8	11	5.6***	3	3 (SDM* 5)	5
*Mutation efficiency in%*	100	100	12	n.r.	55 (SDM* 32)	100
*Max. SSM coverage% (NNK)*	n.r.	n.r.	n.r.	n.r.	n.r.	66–84 (48 clones)
*Unique clones (%)*	n.r.	n.r.	n.r.	n.r.	50 (40 clones)	100 (48 clones)
*Frameshifts, deletions, insertions*	n.r.	n.r.	n.r.	n.r.	n.r.	0
*Restricted in distance between targeted positions* x	No	Yes	Yes	Yes	Yes	No
**Experimental procedure**						
*No. of DNA amplification steps*	2 (b, c)	2 (a, d)	3 (a, b, c)	1 (e)	1 (a)	1 (d)
*Primer modification*	No	5′ phosphorylation	5′ biotinylation	No	5′ phosphorylation	5′ PTO#
*Agarose gel-extraction*	Yes	Yes	Yes	No	No	No
*Endonuclease/Ligase*	No	Yes	Yes	No	Yes	No
*Final cloning step*	Yes	Yes	Yes	No	No	No
*Transformed DNA*	dsDNA	dsDNA	dsDNA	dsDNA	ssDNA	nicked dsDNA

Comparison of key performance parameters and experimental requirements of the most prominent and widely used methods for multi site-directed and site-saturation mutagenesis including the here reported OmniChange method.

6 1158-1160, 1162, 1164-115ties,stuft werden lce ingestuftsay entwickelt werden welcher ein rol (negative control/no primers.

*Site Directed Mutagenesis;

**Site Saturation Mutagenesis;

***45 positions targeted; n.r. (not reported);

x Limitations or restrictions for distance between targeted codons;

# 12 phosphorothioated nucleotides on 5′end; a) Linear DNA amplification b) OEP-PCR c) Assembly/nested PCR d) Standard PCR e) PCR with complementary primers.

Multi site-saturation mutagenesis will likely allow altering of “localizable” properties such as activity and selectivity to identify cooperative amino acid substitutions which would not have been discovered by saturating single codons individually [5], [20] or iteratively [7], [21]. The success of finding such cooperative substitutions highly depends on the quality of the focused mutant library requiring effective and reliable mutagenesis methods as well as complementing screening systems [2], [3].

Here we report a robust, fast and cost-effective method (OmniChange) for simultaneous saturation of five independent codons. OmniChange does not require any enzymes for DNA-modification (restriction, ligation), is fully sequence and fragment size-independent and generates libraries of high genetic diversity in a single PCR step.

## Results

**Figure 1:**
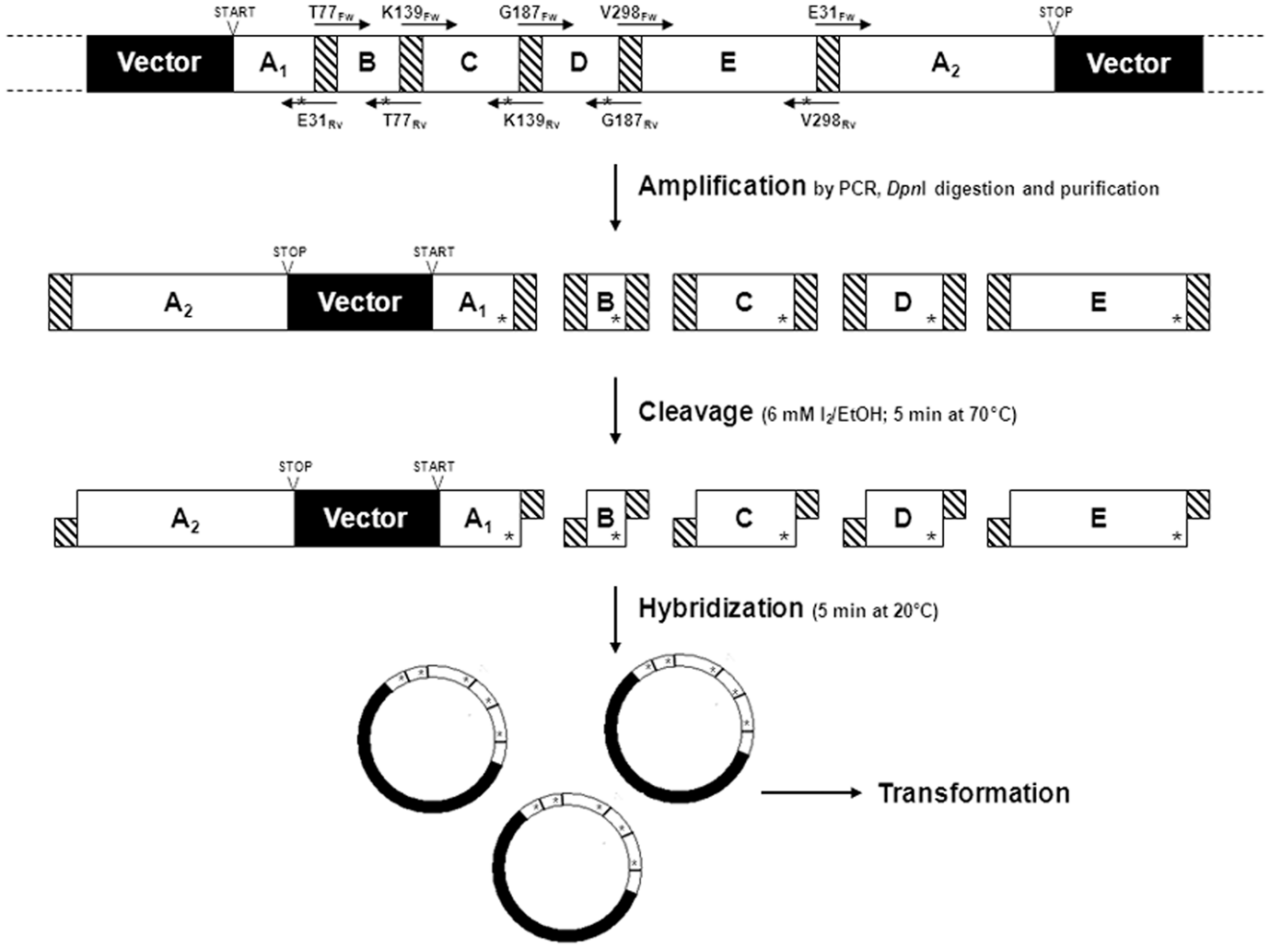
The 4-step strategy for the simultaneous saturation of 5 independent codons by OmniChange. OmniChange comprises four steps: Step 1. Amplification of five DNA fragments bearing a NNK-saturated codon (indicated with *). Step 2. Chemical cleavage to generate complementary single-stranded 5′-overhangs. Step 3. Hybridization of all fragments to a full circular plasmid containing ten DNA nicks. Step 4. Transformation and nick-repair in *E. coli* BL21-Gold (DE3) lacI^Q1^.

The OmniChange multi site-saturation method consists of four steps: Step 1. Fragment generation by PCR with oligonucleotides containing NNK-codons, Step 2. DNA-cleavage reaction for the generation of complementary 5′-overhangs, Step 3. Assembly via DNA-hybridization and Step 4. Transformation and nick repair by *E. coli* BL21-Gold (DE3) lacI^Q1^ (Figure 1).

Amino acid positions E31, T77, K139, G187 and V298 of a phytase from *Yersinia mollaretii* were selected for NNK-saturation. The latter five codons were identified in a directed phytase evolution experiment using the sequence saturation mutagenesis method (SeSaM) [22] for random diversity generation and being screened for improved thermal resistance (unpublished results).

### Preparation of DNA fragments, assembly and transformation into E. coli

Oligonucleotides for the fragment generation (Step 1) can be designed with the basic sequence scheme 5′-(nt)*_12_-NNK-(nt)_12_-3′ having a GC content of at least 40% with a melting temperature (*T*_m_) of preferably 63°C±3°C (calculated using OligoAnalyser tool at http://eu.idtdna.com). The twelve nucleotides on 5′end (nt*) are connected through a phosphorothiodiester bond [23] in contrast to the residual minimal recommended twelve nucleotides on 3′end and NNK codon in the respective reverse primers that are connected through naturally occurring phosphodiester bonds (Table 2). Successful PCR-amplification of the five DNA-fragments A_2_-Vector-A_1_, B, C, D and E (Step 1) was verified by agarose gel electrophoresis (A_2_-Vector-A_1_: 2571 bp; B: 160 bp; C: 199 bp; D: 157 bp and E: 345 bp). After DpnI digestion, iodine cleavage (Step 2), hybridization (Step 3) and transformation into *E. coli* BL21-Gold (DE3) lacI^Q1^ (Step 4) 392 colonies were obtained on a single agar plate assuming a transformation efficiency of 22000 cfu/µg for A_2_-Vector-A_1_ (transformation efficiency of self-made competent cells: 1.4×10^6^ cfu/µg pUC19). Various optimization steps in fragment preparation, cleavage and hybridization were performed until a sufficient amount of colonies (>10000 colonies per µg hybridized DNA) was obtained.

**Table 2:** Oligonucleotides used in the development of OmniChange.

Fragment Amplification	Primer Name	Primer Sequence (5′-3′)
A_2_-Vector-A_1_	E31_Fw_	ctagtgcttcagCGTAAGGGGCAAG
	E31_Rv_	gataaccactcgMNNCAAAGTGTAACCCGTC
B	T77_Fw_	cgagtggttatcTTGAGCCGCCATG
	T77_Rv_	catagaagccgccCATCAGMNNCACTAATTGCGC
C	K139_Fw_	ggcggcttctatgGTGATTATTTCCG
	K139_Rv_	gaaacagtggatcAACCTTMNNCAAATCAGCCTG
D	G187_Fw_	gatccactgtttcACCCCGTCGAAG
	G187_Rv_	cagtgaaattgagAATCTCMNNCATCTGGGCAAATG
E	V298_Fw_	ctcaatttcactgCTTCCCCCTATTGC
	V298_Rv_	ctgaagcactagCGCCGTMNNAATCTGTTGCAAC
C/D/E/A_2_-Vector-A_1_	K139_Fw_	ggcggcttctatgGTGATTATTTCCG
	D52_Rv_	ccacttatccggTGTGACMNNATTCATTAACTCTG
B′	D77_Fw_′	ccggataagtggCCTCAATGGCCGGTAC
	T77_Rv_′	catagaagccgccCATCAGMNNCACTAATTGCGC
Colony PCR	Forward primer	TAATACGACTCACTATAGGG
	Reverse primer	TCCAAAAGAAGTCGAGTGG

Lower case letters indicate phosphorothioated bonds and underlined nucleotides in the reverse primers highlight the NNK degenerated codons.

The following “control experiments” were performed in order to access the versatility and robustness of the developed OmniChange mutagenesis method. First, a possible vector fragment self-hybridization (DNA-fragment A_2_-Vector-A_1_) was determined, yielding four colonies that represent ∼1% of the overall variant number under identical conditions. Secondly, nick repair and assembly to a full length construct (plasmid harboring phytase gene) was verified by DNA-restriction analysis and colony PCR of 20 randomly picked clones showing that complete template DNA information could be assembled by OmniChange with 100% efficiency. Furthermore, five randomly picked clones were sequenced; all five randomized positions were unique and different from the corresponding wild-type codons.

### Obtaining functional proteins from assembled plasmids

As last control, the phytase activity of 20 randomly picked colonies was determined using a 4-methylumbelliferylphosphate detection system [24]. Surprisingly all twenty variants, despite of the high mutational load, showed at least a low but detectable activity proving functional expression from assembled plasmids. Latter controls proved that up to ten nicks can be ligated by *E. coli* BL21-Gold (DE3) lacI^Q1^ and hybridized complementary ends (12 nt overhangs) withstand therefore transformation conditions.

### Statistical analysis of 48 random clones

**Figure 2:**
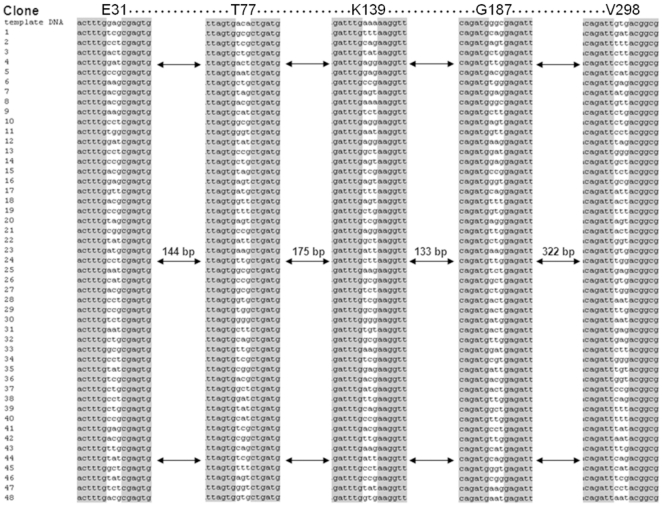
Obtained diversity by OmniChange shown as partial sequence alignment of 48 randomly “picked” clones. Partial sequence alignment of wild-type gene (template DNA) to 48 random clones from a multi site-saturation library generated by OmniChange. The five sites targeted for saturation are highlighted in white. Arrows indicate the distance between the targeted positions in base pairs. A comparison of the codons in the white columns shows the obtained diversity by saturating five independent positions simultaneously by OmniChange.

For detailed statistical analysis, 48 randomly picked clones were sent for forward and reverse sequencing. Multiple sequence alignments with the wild-type sequence confirmed that all five codons were fully saturated and no clone retained the original sequence (Figure 2 and Table 3). Of the 48 sequences analyzed, one wild-type codon could be found at two amino acid positions (E31 and G187) and two wild-type codons were found at position V298. At amino acid position T77 not a single wild-type codon was identified due to the fact that the NNK does not offer the respective wild-type codon [25]. Surprisingly, on position K139 one wild-type codon was identified (clone 8) after sequencing although NNK does not offer AAA as randomly introduced codon (Figure 2). In this case, we have to assume that either oligonucleotide synthesis was not performed completely error-free or exonuclease activity of Phusion polymerase corrected the mismatches during PCR. NNK degeneracy offers a diversity of 32 different codons at each position covering all canonical amino acids and avoids two of the three stop codons [26]. Twenty-one different codons at position E31 could be observed, representing already 65.6% of the possible diversity after sequencing of only 48 clones. For all other positions, even a higher coverage could be obtained (Table 3): T77 (26 unique codons 81.3%), K139 (24 unique codons 75.0%), G187 (27 unique codons 84.4%), V298 (27 unique codons 84.4% diversity). Site-specific cleavage of phosphorothioated nucleotides from 5′-ends of double-stranded DNA was developed as cloning technology reporting that the cleavage reaction did not cause any additional mutations [27]. Sequence analysis of the 48 clones also showed that the cleavage reaction (Step 2) and fragment hybridization (Step 3) did not introduce additional point mutations nor caused insertions or deletions. A mutation frequency of 0.17 per kb (11 mutations in 62.3 kb) was found within the 1.3 kb phytase gene at least 21 bp away from targeted positions which can likely be attributed to the employed polymerase.

**Table 3:** Detailed statistical data of codon diversity generated by OmniChange.

Position	E31	T77	K139	G187	V298
1	aag	aag	aaa	aat	aat
2	aag	aat	aag	aag	aat
3	aat	acg	aag	aag	aat
4	aat	acg	aag	aag	aat
5	acg	act	aat	acg	act
6	acg	agt	acg	acg	act
7	acg	agt	acg	act	agt
8	acg	agt	agg	act	atg
9	acg	atg	agg	act	att
10	acg	att	agg	act	cag
11	acg	cag	agg	agg	cag
12	act	cag	agt	agt	cat
13	atg	cat	agt	agt	cat
14	cat	cat	agt	atg	ccg
15	ccg	ccg	agt	att	cct
16	ccg	ccg	atg	cag	cct
17	ccg	ccg	att	cag	cct
18	ccg	cgg	att	cag	ctg
19	ccg	cgg	cag	cat	ctg
20	cct	ctg	cag	ccg	ctt
21	cct	ctg	ccg	cct	ctt
22	cct	ctg	ccg	cgg	gag
23	cct	ctt	ccg	cgt	gag
24	cct	gat	cct	ctg	gag
25	cct	gcg	ctg	ctg	gag
26	cct	gcg	ctt	ctg	gat
27	cgg	gcg	gag	ctt	gcg
28	ctg	gcg	gag	gag	gct
29	ctg	gct	gtg	gag	ggg
30	ctg	ggg	gcg	gat	ggg
31	gag	ggt	gcg	gat	ggt
32	gag	ggt	gcg	gct	ggt
33	gat	gtg	gct	gct	gtg
34	gat	gtg	gct	ggc	gtg
35	gcg	gtt	ggg	ggt	gtt
36	gct	tag	tat	ggt	tag
37	gtt	tag	tat	gtg	tat
38	tag	tat	tcg	gtt	tcg
39	tat	tat	tcg	Tct	tct
40	tat	tcg	tcg	tgg	tgg
41	tat	tcg	tcg	tgg	tgg
42	tat	tcg	tct	ttg	tgt
43	tcg	tcg	tct	ttg	ttg
44	tcg	tgg	tgt	ttg	ttg
45	tct	ttg	ttg	ttg	ttt
46	tct	ttg	ttg	ttg	ttt
47	tgg	ttt	ttt	ttg	ttt
48	ttg	ttt	ttt	ttt	ttt
**Different codons**	**21**	**26**	**24**	**27**	**27**
**Maximal diversity (NNK)**	**32**	**32**	**32**	**32**	**32**
**Obtained diversity [%]**	**65.6**	**81.3**	**75.0**	**84.4**	**84.4**

### Modular assembly of sublibraries with 2 and 4 simultaneous saturated positions

Furthermore, OmniChange allows a modular and combinatorial assembly of the targeted codons to study additive and cooperative effects in detail and for any targeted position combination. With the same sets of oligonucleotides position D52 was selected to investigate whether codons with less than 100 bp distance to each other could be mutated. As proof of principle, two libraries were generated: library 1 targeting positions D52 and T77 (primer combinations K139_Fw_/D52_Rv_ (fragment C-D-E-A_2_-Vector-A_1_) and T77_Fw_′/T77_Rv_ (fragment B′); see Table 2) and library 2 targeting T77, K139, G187 and V298 (oligonucleotide combinations in Table 2 yielded fragments C, D, E and A_2_-Vector-A_1_-B). Sequencing of five randomly picked clones from both libraries showed again that targeted codons were saturated exclusively whereas all other positions remained unchanged (data not shown).

## Discussion

The research fields of rational and semi-rational protein engineering are key drivers for improving protein properties such as activity and selectivity through iterative rounds of hypothesis generation and focused mutagenesis [28]. Diversity generation in focused mutagenesis studies is usually performed by site-directed mutagenesis in which single positions are either specifically changed or saturated [2]. Multi site-directed mutagenesis methods hold the promise to discover synergistic amino acid substitutions, opening novel and perhaps better approaches for rational and semi-rational protein engineering [5]. However, state of the art screening technologies allow efficient sequence space exploration of mutant libraries with up to five simultaneously NNK-saturated codons (3.2×10^6^ different protein variants; 3.4×10^7^ different codon variants). For example, assuming that a suitable flow cytometry screening is available, sampling of up to 10^8^ variants [29] would completely cover the theoretical sequence space of five simultaneously NNK-saturated codons with a confidence level of 99.6% [25]. In contrast, only 52% of the generated protein sequence space can be explored by screening 10^8^ variants when six codons are NNK-saturated. Therefore, OmniChange was designed for the simultaneous saturation of five independent codons with the possibility of a modular and combinatorial assembly of the targeted positions (two to four) to study additive and cooperative effects in detail and for each position (see results section). In this publication we report and address challenges in DNA-fragment preparation (DNA concentration and cleavage mix optimization), and DNA-hybridization (conditions and sequence of fragment addition) during the development of OmniChange. In addition, it was unknown whether the five assembled DNA fragments (Figure 1, Step 3) could maintain their hybridization and specific order during heat shock treatment and subsequent transformation (Step 4). The ability of the *E. coli* host to repair up to ten nicks in very close distance (<100 bp) without insertions or deletions was another important aspect to be taken into consideration during development of the OmniChange method.

A detailed overview and performance comparison on focused mutagenesis methods is provided in several reviews [2], [3]. Table 1 compares the most prominent, advanced or “best” characterized multi-site mutagenesis methods according to key performance parameters and experimental set ups. The methods OD SPM and POEP were included in Table 1 due to their conceptual novelty and high number of simultaneously targetable sites despite that only site-directed mutagenesis studies were reported [12], [15]. Both methods are not designed with the intention to screen large number of variants and would have to be optimized for saturation mutagenesis and cloning efficiency for instance by avoiding a gel extraction step. The ISOR method maximizes diversity in a focused mutant library by adding 45 mutagenic oligonucleotides to a single multiplex assembly PCR obtaining an average of 5.6 substitutions per gene [19]. ISOR seems from our point of view to be more attractive for directed evolution experiments than for systematic studies of up to five sites in semi-rational protein engineering experiments since the targeted sites are statistically incorporated. However the design, cooperative work and fine tuning of 45 primers in a multiplex PCR remains the major challenge in the ISOR method. The CASTing method relies on the robust and widespread Mutagenic Plasmid Amplification protocol [6], [11] and was used to saturate two to three codons in the binding pocket per round [30]. As a prerequisite for saturation, mutated codons have to be located on the same primer which limits the distance between two simultaneously saturated codons from 9 to 12 bp. ISM expanded the CASTing concept to sites not located in the binding pocket and advanced the concept by introducing iterative rounds of saturation mutagenesis and screening [13]. A combination of ISM and CASTing was reported as iterative CASTing focusing on the mutagenesis of residues in the substrate binding pocket in iterative cycles of diversity generation and screening [7]. Although ISM minimizes the oversampling and screening efforts, the cooperative interactions between genetically far distant amino acids cannot be investigated entirely since only improved variants are chosen for subsequent rounds of mutagenesis. The QuikChange Multi Site-Directed Mutagenesis Kit (Stratgene, La Jolla, CA, USA) and the OmniChange method differ fundamentally from the CASTing and ISM approaches by employing multiple non-complementary mutagenic primers in a single DNA amplification round which allows targeting of multiple distant codons at the same time [18]. Hogrefe et al. described in detail the major limitations of the QuikChange Multi Site-Directed Mutagenesis Kit which represents a very well suited method for saturating up to three independent codons. From three sites targeted, only 55% of forty clones sequenced turned out to have all three codons mutated with nearly all codons being unique (Table 1) [18]. However, expanding the protocol to more than three codons and/or reducing the distance between the focused positions would be significantly limited by increasing likeliness for undesired oligonucleotides interactions [16], and by an inefficient and concerted action of the employed thermostable ligase and polymerase during each cycle of the multiplex PCR amplification [17]. In general, methods for simultaneous saturation mutagenesis of multiple codons often rely on several PCR steps accompanied by DNA modification steps (Table 1). Targeting more codons would increase the number of required PCRs (unwanted additional mutations) and DNA modification steps; the latter renders these mutagenesis methods more and more tedious and less efficient [14],[18].

OmniChange solved all technical challenges in saturating five codons simultaneously in a four step method (Figure 1) which can be accomplished in a single day. Sequence analysis of 48 randomly picked clones of a phytase model library (simultaneous NNK-saturation of five sites) showed that up to 27 of 32 possible codons (84.4% of the diversity offered) could be identified at individual positions (Table 3). The overall distribution of different codons was unbiased, since no codon was preferentially integrated on targeted amino acid positions (Table 3). All randomly picked clones were unique and contained full-length and functional plasmids with a transformation efficiency of 2.2×10^4^ cfu per µg A_2_-Vector-A_1_ using homemade chemically competent *E. coli* BL21-Gold (DE3) lacI^Q1^ cells (1.4×10^6^ cfu per µg pUC19). However, even if highly competent cells with transformation efficiencies of up to 10^9^ cfu per µg would be used, one would generate theoretically ∼10^7^ variants which is still one order of magnitude lower than the 10^8^ variants required to fully sample the protein sequence space generated by five NNK-codons [25]. Since it cannot always be foreseen which of the targeted positions is beneficial or leads to fully inactivation of the mutated protein we investigated the possibility to saturate only two or four codons at the same time using the same set of oligonucleotides. With the same OmniChange protocol we could generate “sub-libraries” without re-designing or ordering additional oligonucleotides which makes OmniChange a versatile and modular diversity generation method matching the throughput of the employed screening system. The transformation of nicked DNA and the latter repair by the host organism (*E. coli*) is commonly regarded as a crucial and limiting factor for fusion of two or more DNA fragments [31]. Enzymatic ligation of nicked DNA became a standard approach during cloning experiments with the aim to make transformation more efficient. OmniChange enables transformation of circular DNA fragments with up to ten DNA nicks with an excellent efficiency underlining the exceptional potential of ligase-free or enzyme-free methods in genetic engineering. In future, chemical gene synthesis might develop into an attractive option for multi site-saturation mutagenesis libraries reducing the challenges of oversampling in highly diverse libraries (>five randomized codons) [32], [33]. Employing phosphorothiolated oligonucleotides for OmniChange will always be more economical and faster compared to automated gene synthesis facilities. Currently chemical DNA synthesis methods are at least one order of magnitude more expensive, employ expensive DNA synthesizers, and are still DNA-fragment size-restricted for error-free synthesis [34].

Rational and semi-rational protein design through focused multi site-saturation mutagenesis enables fine tuning of enzyme properties to industrial demand by improving e.g. enzyme selectivities, activities, catalytic promiscuity and thermostability. OmniChange is a robust, time-efficient and fully sequence-independent multi site-saturation mutagenesis method which addresses the actual and important demands in enzyme catalysis and protein engineering. In addition, it is for the first time possible to study systematically and in a modular manner cooperative effects through multiple site substitution of amino acids which are located closely to each other in the 3D protein structure. On the gene level the latter amino acid positions can however be distant from each other. Two further studies in our group prove that OmniChange generates substitutions with cooperative effects that would have been missed by selecting the most active variants in an iterative site saturation mutagenesis experiment (unpublished data). OmniChange will very likely allow saturating more than five codons simultaneously or implementing primers with multiple saturated codons in Step 1 (Figure 1) in order to saturate codons which are close to each other on nucleic acid sequence. Saturating more than five positions at the same time is however not desired in directed evolution experiments due to limitations in throughput of screening technologies which do not imply selection systems. OmniChange showed further that twelve complementary nucleotide overhangs are sufficient to fuse five DNA fragments in a predefined order without frameshifts, deletions or additional enzymatic treatment (Figure 1). Interestingly, the multi nicked plasmid DNA (10 nicks) was efficiently transformed into an *E. coli* host outperforming enzymatic based strategies by its robustness and simplicity in handling. Another important fact during development of OmniChange was the oxidizing potential of iodine/EtOH in repect to secondary mutations within the generated library. Eckstein and Gish reported in detail the introduction of phosphorothiolated nucleotides in DNA and RNA and the benefits of the phosphorthioate modification for sequencing applications [35]. The proposed mechanism of cleavage of phosphorothiolated nucleotides through iodine/EtOH alkylation was reported to be efficient and regiospecific for nucleotides being connected through phosphorothiodiesterbonds [23]. Within the generated OmniChange library only a few transition mutations were obtained away from targeted sites that can be attributed to the employed polymerase showing that exclusively phosphorothiolated nucleotides are oxidized by iodine/EtOH. In addition, the recently PLICing method applies phosphorothiolated nucleotides that are cleaved with I_2_/EtOH with a very low mutation frequency (1 transition in 9 kbp) [27].

OmniChange is in essence a conceptually novel and powerful method for multi site-saturation mutagenesis which will equip scientists with exciting opportunities to study the influence of synergistic amino acid substitutions in semi-rational protein engineering campaigns. We hope that protein engineers will use the OmniChange method for generating libraries of high diversity with minimum efforts to explore the potential of the natural protein sequence space efficiently.

## Materials and Methods

### Enzymes, reagents and oligonucleotides

All reagents used were of analytical grade and purchased from Sigma-Aldrich (Steinheim, Germany) and AppliChem (Darmstadt, Germany). Enzymes were obtained from New England Biolabs (Frankfurt, Germany), and dNTPs were purchased from Fermentas (St.Leon-Rot, Germany). Oligonucleotides were synthesized at HPLC-purity by Eurofins MWG Operon (Ebersberg, Germany) in salt-free form. According to suppliers recommendations all oligonucleotides were diluted in Milli-Q water to a final concentration of 100 µM. All oligonucleotides used in this study are summarized in Table 2.

### Cell strains and vectors

The 1.3 kb phytase gene from *Yersinia mollaretii* (GenBank accession no. JF911533; Release date 1^st^ September 2011) was cloned and expressed in a pET-22b(+)-derived Novagen vector (Darmstadt, Germany) named pALXtreme-5b (2.1 kb) [27]. Gene expression from pALXtreme-5b requires *Escherichia coli* BL21-Gold (DE3) lacI^Q1^ as host system due to necessary incorporation of the lacI repressor gene into the bacterial genome [27]. Chemically competent *E. coli* BL21-Gold (DE3) lacI^Q1^ cells were prepared according to a standard protocol [36] with a determined transformation efficiency of 1.4×10^6^ cfu/µg pUC19.

### Mutagenic oligonucleotide design

**Figure 3:**
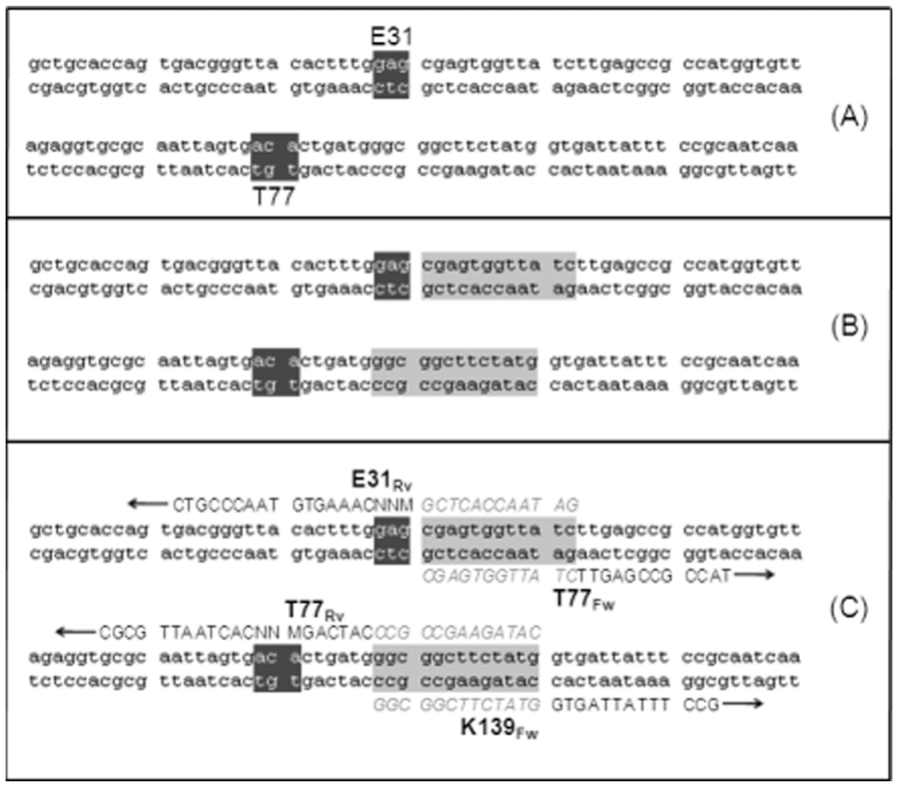
Three steps in the primer design for the OmniChange method on the examples of positions E31 and T77. (A) Selection of targeted codons for NNK saturation (dark-grey highlighted letters). (B) Twelve nucleotides downstream of a targeted codon are selected as phosphorothiolated nucleotides for subsequent overhang generation by chemical cleavage into multiple small fragments. Remaining overhangs enable efficient hybridization of generated DNA fragments (light-grey highlighted letters). (C) Design of each oligonucleotide 3′end for successful PCR amplification of DNA fragments. Arrows indicate Phusion DNA Polymerase amplifcation direction. Italic letters are the phosphorothiolated nucleotides on the 5′ends of every primer used in the OmniChange method.

Ten oligonucleotides were designed as PCR-primers for simultaneously saturating five codons (Table 2). Generally, oligonucleotides for OmniChange PCR should be designed to maximize product yields in PCRs (e.g. minimum 40% GC content) [17]. Reverse primers harboring a NNK codon were designed to have a GC content of minimum 40% and a *T*_m_ of preferably 63°C±3°C (calculated using OligoAnalyser tool at http://eu.idtdna.com). In addition, all oligonucleotides have at least 12 or 13 phosphorothioate nucleotide bonds at the 5′-terminus to generate single stranded overhangs for DNA hybridization after iodine cleavage [27] (Table 2). A detailed guidance for oligonucleotides design is described exemplary for positions E31 and T77 in Figure 3.

### PCR amplification of DNA fragments

Standard plasmid isolation was accomplished by using the QIAGEN QIAprep Spin Miniprep Kit (Hilden, Germany). A standard PCR set up contained 1x Phusion DNA polymerase buffer, 0.2 mM dNTP mix, 0.4 µM fragment specific forward and reverse primer (Table 2), 5 U Phusion DNA polymerase and 20 ng of DNA template in a final volume of 50 µl. All PCR amplifications were carried out in an Eppendorf Mastercycler proS (Hamburg, Germany) using thin-wall PCR tubes (Sarstedt, Nuembrecht, Germany). A PCR for targeted codons was performed by pre-heating each fragment sample mixture to 94°C for 3 min followed by 25 cycles of 94°C for 30 s, 55°C for 30 s and 72°C for 30 s (fragments B, C, D and E) or 1.5 min (fragment A_2_-Vector-A_1_). For final elongation a fill-up cycle was performed at 72°C for 1 min (fragment B, C, D and E) or 3 min (fragment A_2_-Vector-A_1_). PCR products were separated on 2% (w/v) Tris-acetate-EDTA (TAE) agarose gels to confirm the size of the respective PCR products [37]. Specific degradation of methylated template DNA was realized by adding 1 µl (20 U) of DpnI to each PCR sample. After incubation (37°C for 4 h) the PCR products were purified using the Macherey-Nagel NucleoSpin ExtractII DNA purification kit (Dueren, Germany) and eluted in 50 µl Milli-Q water. Purified PCR products were quantified using a Nanodrop 1000 UV spectrophotometer (NanoDrop Technologies, DE, Wilmington, USA).

### Iodine cleavage, DNA-fragment annealing and transformation

All steps were performed on ice unless stated otherwise. Purified fragment A_2_-Vector-A_1_ and fragments B, C, D, and E were diluted to 0.02 pmol/µl or 0.11 pmol/µl respectively using Milli-Q water. The PCR-products were cleaved according to the PLICing cloning technology [27] with the following alterations: 4 µl of each PCR fragment were combined with 2 µl iodine cleavage buffer (0.25 M Tris-HCl pH 9.0; 30 mM iodine; 30% (v/v) ethanol) in PCR tubes, incubated in a thermocycler (70°C; 5 min) and finally kept on ice (5 min). Due to low solubility of crystalline iodine in ddH_2_O, a stock of 100 mM iodine solved in 100% ethanol was prepared and diluted to the employed concentration within the iodine cleavage buffer. Annealing of cleaved fragments was performed gradually by first adding 6 µl fragment B cleavage mix to 6 µl of fragment A_2_-Vector-A_1_ cleavage mix. Hybridization of both fragments was performed at 20°C for 5 min. Subsequently, cleavage mixtures of fragments C, D and E were supplemented (gradually with 5 min incubation between addition of each fragment) and hybridized, yielding a circular and nicked B-C-D-E-A2-Vector-A1 construct that requires in total an assembly time of 20 min. The mixture was kept on ice for further 5 min. Self-hybridization of the A_2_-Vector-A_1_ fragment was determined by preparing solely the A_2_-Vector-A_1_ fragment identically to the nicked B-C-D-E-A_2_-Vector-A_1_ construct. In the latter quality control, fragments B, C, D and E were replaced each by 4 µl Milli-Q water. Finally, 4 µl of both hybridization mixtures were transformed via heat shock and without any further purification step into 100 µl chemical competent *E. coli* BL21-Gold (DE3) lacI^Q1^ cells [38]. Transformation mixtures were spread on agar plates (LB; 100 µg/ml ampicillin) and incubated overnight.

### Colony PCR and sequencing of plasmid constructs

Colony PCRs were performed to verify the correct assembly of five DNA fragments to the B-C-D-E-A_2_-Vector-A_1_ construct (Figure 1) carrying five saturated codons. Twenty colonies were randomly picked into liquid LB media (2 ml, 100 µg/ml ampicillin) and grew overnight (14 h, 37°C, 250 rpm). Liquid cultures (10 µl) were lysed (50 µl Milli-Q water, 95°C, 15 min), centrifuged (13000 g, 2 min) and 5 µl of clear supernatant served as template DNA for colony PCR-amplifications (1x Taq DNA polymerase buffer, 0.2 mM dNTP mix, 0.4 µM Forward and Reverse primer (Table 2), 5 U Taq DNA polymerase; identical cycling program as for fragment A_2_-Vector-A_1_ amplification). Elongation times in each PCR cycle were increased to 1 min and to 3 min in the fill up reaction. PCR samples were separated on 1% (w/v) TAE agarose gels to verify the size of each PCR product. Sequencing was performed at GATC Biotech (Konstanz, Germany) and sequence analysis was carried out using Clone Manager 9 Professional Edition software (Scientific & Educational Software, Cary, NC, USA).

### Phytase activity measurements in microtiter plates

Phytase activity determination was performed on randomly picked colonies, which were transferred from agar plates into a Greiner Bio-One 96-well microtiterplate (Greiner, Frickenhausen, Germany) filled with 200 µl sodium acetate (NaAc) buffer (pH 5.5, 250 mM sodium acetate; 1 mM CaCl_2_; 0.01% (w/v) Tween20). Enzymatic reactions were initiated by adding 50 µl NaAc buffer containing 4-methylumbelliferylphosphate (1 mM). Phytase activity was determined by following the fluorescence increase due to 4-methylumbelliferone formation (λ_ex_: 360 nm; λ_em_: 460 nm) [24] using an Infinite M 1000 plate reader (Tecan, Groeding, Austria, 37°C for 15 min). Colonies with the original (template) phytase gene and colonies only carrying the empty pALXtreme-5b vector were used as reference.
